# Frequency of Positive Antinuclear Antibody in People With Active Tuberculosis and Its Changes With Antitubercular Therapy

**DOI:** 10.7759/cureus.81116

**Published:** 2025-03-24

**Authors:** Naznin Naher, Hasan Imam, Sunil Kumar Biswas, Tonmoy Biswas, Mak Azad, Md Nazmul Hasan

**Affiliations:** 1 Department of Internal Medicine, Bangabandhu Sheikh Mujib Medical University, Dhaka, BGD; 2 Department of Internal Medicine, Green Life Medical College and Hospital, Dhaka, BGD

**Keywords:** active tuberculosis, antinuclear antibody, antitubercular therapy, autoimmunity, immunofluorescence pattern

## Abstract

Background

Tuberculosis (TB), one of the most prevalent chronic infectious diseases in our country, can disrupt immune function and induce autoantibodies such as antinuclear antibodies (ANAs). As ANA positivity occurs in both TB and autoimmune diseases such as systemic lupus erythematosus (SLE), it can complicate the diagnosis when symptoms overlap. Furthermore, it remains unclear whether ANA positivity resolves with successful TB treatment. This study aimed to assess the prevalence of ANA in active TB patients and evaluate changes in ANA status following anti-TB therapy.

Methods

This prospective observational study was conducted at the Bangabandhu Sheikh Mujib Medical University (BSMMU) and included 150 adult patients (≥18 years) with newly diagnosed active tuberculosis and no history of autoimmune diseases. ANA testing was performed at baseline, and repeat ANA testing was conducted after three and six months of anti-TB therapy. Qualitative data were summarised as numbers and percentages, with chi-square, t-test, and Mann-Whitney U applied as appropriate.

Results

A total of 13 out of 150 patients (8.7%) with active tuberculosis tested positive for ANAs. Among the 53 patients with pulmonary TB, six (11.3%) were ANA positive, while seven out of 97 patients (7.2%) with extrapulmonary TB tested ANA positive. The most common ANA pattern observed was the coarse speckled pattern, found in nine of the 13 ANA-positive patients (69.23%). After six months of antitubercular therapy, 12 out of the 13 initially ANA-positive patients (92.3%) became ANA negative.

Conclusion

Positive ANA antibodies were detected in pulmonary TB patients as well as in extrapulmonary TB patients. However, no significant associations were found between antinuclear antibodies and active tuberculosis.

## Introduction

Tuberculosis (TB) is one of the world's most persistent chronic bacterial infections, affecting an estimated 10.6 million individuals globally in 2022. The 30 countries with the highest TB burden accounted for more than 87% of newly diagnosed cases of TB; these countries included Bangladesh, China, the Democratic Republic of the Congo, India, Indonesia, Nigeria, Pakistan, and the Philippines, which together accounted for more than two-thirds of all cases worldwide [1].‌ In Bangladesh, the estimated incidence rate for all forms of tuberculosis in 2021 was 221 per 100,000 people. In the Dhaka metropolitan area, there were 12760 new extrapulmonary TB (EPTB) cases and 7373 new pulmonary TB (PTB) cases in 2021 [2]. TB is a multifaceted disease that affects multiple organs and often presents with a spectrum of symptoms that can closely resemble those observed in autoimmune diseases [3]. Autoimmune disorders are hallmarks of a wide range of diseases that occur due to pathological immune responses to self-antigens. It is postulated that certain chronic infections, such as tuberculosis, can initiate the development of autoantibodies through molecular mimicry and persistent host responses [4]. Numerous studies have revealed the presence of various autoantibodies, such as antinuclear antibodies (ANA), anti-double-stranded DNA (anti-dsDNA), anti-Smith (Sm), and anti-ribonucleoprotein (anti-RNP), in the serum of active TB patients. Notably, these autoantibodies, which typically serve as diagnostic markers for autoimmune diseases, have also been detected in individuals with TB. Among autoantibodies, ANA serves as a key screening test for autoimmune diseases [5]. ANAs are a class of autoantibodies that are associated with and used in the diagnosis of autoimmune diseases of the connective tissue, such as mixed connective tissue disease, systemic lupus erythematosus (SLE), and systemic sclerosis [6]. As the symptoms of tuberculosis often mimic those of SLE, including fever, malaise, and weight loss, clinicians may order autoimmune serology for diagnosis. When autoimmune markers become positive, it can create a diagnostic dilemma, complicating the differentiation between the two conditions [7]. Although autoantibodies are common in active TB patients, most of the research is cross-sectional and provides limited insights into the dynamic changes in these autoantibodies following anti-TB therapy. This study aimed to determine the frequency of ANA positivity in active TB patients and assess whether it turned negative after antitubercular therapy. 

This article was previously presented as a meeting abstract at the 33rd Annual Conference and International Scientific Seminar (APBCON), held on May 31, 2024, in Dhaka, Bangladesh.

## Materials and methods

Study design and setting

This prospective observational study was conducted in the Department of Internal Medicine at Bangabandhu Sheikh Mujib Medical University (BSMMU), Dhaka, Bangladesh, over 17 months from September 2022 to March 2024. Participants were recruited from BSMMU, Dhaka Medical College, and the KMSS DOTS Center, Abul Hasnat Road, Dhaka. Ethical approval was obtained from the Institutional Review Board (IRB) at BSMMU.

Sample size and selection criteria

A total of 150 patients with a diagnosis of active TB were included in this study. To achieve 90% power with a significance level of 0.05, a sample size of 141 was determined using a two-sided Z-test. The study assumes that the anticipated population proportion under the null hypothesis (P0) is 20%, and the prevalence of autoantibody in patients with active tuberculosis is 32% [6]. Patients were recruited through non-probability convenience sampling.

Inclusion criteria

Patients aged 18 years or older who were diagnosed with active tuberculosis were included in the study. Pulmonary TB patients were recruited based on bacteriological confirmation through microscopy, culture, or GeneXpert. Extrapulmonary TB patients were included based on diagnoses established through suggestive clinical features and radiological evidence, microbiological testing, histopathological analysis, biochemical or immunological assessments, or molecular methods [8].

Exclusion criteria

Patients with autoimmune diseases, HIV, malignancy, or chronic respiratory diseases were excluded. Those with extrapulmonary TB requiring steroids, relapse, multidrug-resistant (MDR)/extensively drug-resistant (XDR) TB, or meningeal TB were not included. Patients on anti-TB therapy for more than seven days, using oral contraceptive pills (OCPs), steroids, or disease-modifying antirheumatic drugs (DMARDs), as well as pregnant and lactating women, were excluded.

Study procedure

Patients were initially informed about the study's objectives and the assurance of confidentiality. Written informed consent was obtained. Sociodemographic data and medical history were collected through in-person interviews and recorded in a data sheet. A 5 ml venous blood sample was drawn from the antecubital vein, and serum was prepared for autoantibody detection. ANA was estimated using indirect immunofluorescence on Hep-2 cells, according to the manufacturer's guidelines on the autoanalyzer at the Department of Microbiology and Immunology at BSMMU. The results were recorded in the data sheet. ANA-positive patients were clinically evaluated for improvement and monitored for new symptoms or adverse drug reactions. Follow-up serum samples were collected from an ANA-positive patient at the end of three and six months of anti-tubercular therapy to assess the effect of treatment on ANA positivity. These follow-up samples were collected at the BSMMU DOTs corner, and patients received phone counseling and linkage.

All TB patients were administered daily doses of isoniazid (INH), rifampin (RIF), ethambutol (ETM), and pyrazinamide for the first two months, followed by daily doses of RIF and INH for the next four months. The primary care physician could adjust the regimen if necessary. All ANA-positive patients in this study had an uneventful recovery following anti-tubercular chemotherapy.

Laboratory methods

Three milliliters of whole blood were collected from each participant in a red-top vacutainer and transported with an ice pack to the laboratory, where it was centrifuged, and the serum was stored at −80°C until analysis. ANA determination was performed via the ANA Hep-2 Plus indirect immunofluorescence test, which detects antinuclear antibodies by incubating diluted serum with Hep-2 cell antigens, labeling with fluorescent anti-IgG, and examining specific staining patterns under a fluorescence microscope, which has been performed in the microbiology department of BSMMU.

Statistical analysis

After the data were cleaned and checked, qualitative data were expressed as numbers and percentages. The quantitative data are presented as the means and standard deviations. The level of significance was set at 5%, and a p-value <0.05% was considered significant. The chi-square test, independent t-test, and Mann-Whitney U test were applied for the analyses of qualitative and quantitative variables, as appropriate. All analyses were performed via SPSS software (version 26.00; IBM Corp., Armonk, NY, USA).

## Results

A total of 150 active tuberculosis patients were included in the study, with 97 cases of extrapulmonary tuberculosis and 53 cases of pulmonary tuberculosis. ANA testing revealed that 13 patients were positive, whereas the remaining 137 tested negative. The clinicodemographic characteristics of ANA-positive and ANA-negative patients are summarised in Table 1.

**Table 1:** Clinicodemographic characteristics of the ANA-positive and ANA-negative study subjects a = Pearson chi-square test, b = Fisher’s exact test, c = Mann-Whitney U test ANA: antinuclear antibody, PTB: pulmonary tuberculosis, EPTB: extrapulmonary tuberculosis, SD: standard deviation p-value of <0.05 is considered statistically significant

Characteristics of patient	Category	ANA results in active TB patient		
		ANA negative (N = 127)	ANA positive (N = 13)	p value
Age year (mean ± SD)		35.98 ± 15.8	30.15 ± 8.325	0.413^c^
Age range (years)	18-34	75 (54.7%)	8 (61.5%)	0.157^b^
	35-51	34 (24.8%)	5 (38.5%)	
	>51	28 (20.4%)	0	
Gender	Male	78 (56.9%)	6 (46.2%)	0.560^a^
	Female	59 (43.1%)	7 (53.8%)	
Duration of symptoms months (mean ± SD)		2.154 ± 1.772	1.774 ± 1.697	0.368^c^
Duration of symptoms	<2 month	77 (56.2%)	6 (46.2%)	0.500^b^
	2-5 month	53 (38.7%)	6 (46.2%)	
	>5 month	7 (5.1%)	1 (7.7%)	
Fever	YES	94 (68.6%)	9 (69.2%)	0.736^b^
	NO	43 (31.4%)	4 (30.8%)	
Weight loss in kg (mean ± SD)		4.80 ± 4.6	6.00 ± 4.8	0.267^c^
Weight loss	≤5 kg	99 (72.3%)	7 (53.8%)	0.203^b^
	>5 kg	38 (27.7%)	6 (46.2%)	
Malaise	YES	71 (51.8%)	7 (53.8%)	0.94^b^
	NO	66 (48.2%)	6 (46.2%)	
Arthralgia	YES	22 (16.1%)	5 (38.5%)	0.127^b^
	NO	114 (83.2%)	8 (61.5%)	
Myalgia	YES	11 (8.0%)	4 (30.8%)	0.40^b^
	NO	125 (91.2%)	9 (69.2%)	
Sites of tuberculosis	PTB	47 (34.30%)	6 (46.2%)	0.393^a^
	EPTB	90 (65.7%)	7 (53.8%)	
Sites of extrapulmonary TB	Bone TB	14 (10.2%)	1 (7.7%)	0.626^a^
	Genital TB	2 (1.5%)	0	
	Tuberculous ascites	2 (1.5%)	1 (7.7%)	
	Lymph node TB	36 (26.3%)	5 (38.5%)	
	Tuberculous pleural effusion	23 (16.8%)	0	
	Skin TB	5 (3.6%)	0	

Clinicodemographic characteristics of ANA-positive and ANA-negative subjects

The mean age of the participants (n = 150) was 35.47 ± 15.36 years, with ANA-negative patients (n = 137) having a mean age of 35.98 years, whereas ANA-positive patients (n = 13) had a lower mean age of 30.15 ± 8.33 years. The majority of both ANA-positive (8/13, 61.5%) and ANA-negative (75/137, 54.7%) subjects belonged to the 18-34 age group. In the ANA-positive group, the majority (53%) were female, whereas in the ANA-negative group, the majority (56%) were male. There was no significant difference in the duration of symptoms between the ANA-negative group (2.154 ± 1.772) and the ANA-positive group (1.774 ± 1.697) (p = 0.368). Weight loss was higher in the ANA-positive group (6.00 ± 4.8) compared to the ANA-negative group (4.80 ± 4.6). Fever was the most prevalent symptom in both groups, with no significant difference between them, occurring in 69.2% of ANA-positive and 68.6% of ANA-negative patients. Malaise was reported by nearly half of the patients in both groups, while arthralgia and myalgia were less common. Significant weight loss (>5 kg) was observed in six (46.2%) ANA-positive patients compared to 38 (27.7%) ANA-negative patients (Table 1). In the ANA-negative group, 47 (34.3%) had PTB, while 90 (65.7%) had EPTB. In the ANA-positive group, six (46.2%) had PTB, while seven (53.8%) had EPTB. Among both ANA-positive and ANA-negative patients, lymph node TB was the most common form of EPTB, affecting five (38.5%) ANA-positive patients and 36 (26.3%) ANA-negative patients, respectively.

ANA immunofluorescent pattern among ANA-positive study subjects

**Figure 1:**
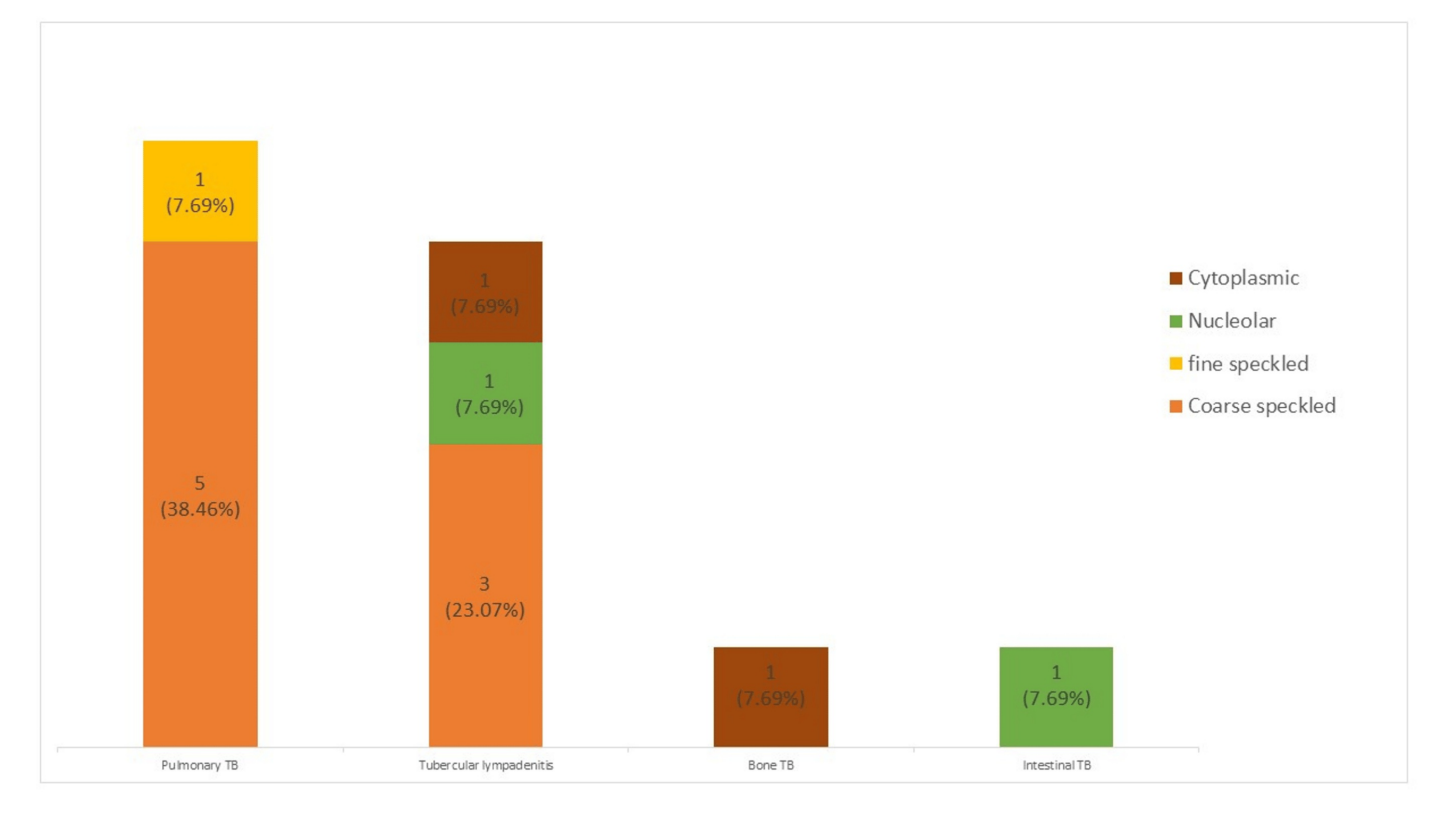
ANA patterns among pulmonary and extrapulmonary tuberculosis patients TB: tuberculosis, ANA: antinuclear antibodies The table presents the ANA immunofluorescence patterns (homogeneous, speckled, or nucleolar) classified based on the fluorescence observed

Figure 1 illustrates the ANA immunofluorescent pattern among ANA-positive study subjects (n = 13). Among pulmonary tuberculosis patients, 5/13 (38.46%) exhibited a nuclear coarse speckled pattern, while 1/13 (7.69%) demonstrated a fine speckled pattern. Among those with extrapulmonary tuberculosis, tubercular lymphadenitis had the highest incidence of positive ANA, with patterns including coarse speckled ANA in 3/13 (23.07%), nucleolar ANA in 1/13 (7.69%), and cytoplasmic ANA in 1/13 (7.69%) patients. Additionally, one patient with bone tuberculosis (7.69%) displayed a cytoplasmic ANA pattern, while another with intestinal tuberculosis (7.69%) exhibited a nucleolar ANA pattern.

ANA test results with antitubercular chemotherapy

Table 2 summarises the follow-up of 13 ANA-positive patients at the end of the third and sixth months of anti-TB therapy. By three months, 8/13 (61.5%) transitioned to ANA-negative status, whereas 3/13 (23.07%) remained weakly positive, 1/13 (7.7%) moderately positive, and 1/13 (7.7%) strongly positive. Among those with persistent ANA positivity at three months, follow-up at six months revealed that most 12/13 (92.3%) transitioned to ANA-negative, with only one patient remaining ANA-positive after six months of anti-tubercular therapy. This patient was monitored for one year for clinical signs of connective tissue disease, but no symptoms developed.

**Table 2:** ANA test results with antitubercular chemotherapy (n=13) ANA immunofluorescence patterns were monitored before and after anti-TB therapy. A shift from moderately/weakly positive patterns to negative results was observed over time. TB: tuberculosis, ANA: antinuclear antibodies

ANA Result	ANA in first visit (Before anti-TB therapy)	ANA in second visit (After three months of anti-TB therapy)	ANA in third visit (After six months of anti-TB therapy)
Strongly positive	1 (7.7%)	1 (7.7%)	1 (7.7%)
Moderately positive	11 (84.4%)	1 (7.7%)	0
Weakly positive	1 (7.7%)	3 (23.07%)	0
Negative	0	8 (61.5%)	12 (92.3%)
Total	13	13	13

Factors associated with autoantibody positivity

Logistic regression analysis was used to evaluate the associations between various factors and the likelihood of ANA positivity among TB patients. The analysis revealed no statistically significant predictors of ANA positivity. Variables such as age (p = 0.237), sex (p = 0.420), symptom duration (p = 0.588), fever (p = 0.236), weight loss (p = 0.345), malaise (p = 0.389), arthralgia (p = 0.499), and myalgia (p = 0.118) did not demonstrate significant associations. These results are presented in Table 3.

**Table 3:** Multivariate logistic regression analysis to predict factors associated with autoantibody positivity The table presents the results of logistic regression analysis to evaluate factors associated with ANA positivity in patients. For each factor, the B coefficient represents the regression estimate, and the standard error is the standard error of the B coefficient. The odds ratio (OR) reflects the odds of ANA positivity associated with each factor, with values greater than 1 suggesting a positive association and values less than 1 indicating a negative association. A P value less than 0.05 is typically considered statistically significant. CI: 95% Confidence Interval, ANA: antinuclear antibodies

Factors/Variable						
ANA positive	B	Std. Error	P value	Odds ratio	95% CI for OR	
					Lower	upper
Age	-0.028	0.024	0.237	0.972	0.928	1.019
Sex	0.522	0.648	0.420	1.685	0.474	5.996
Duration of symptoms in months	0.099	0.183	0.588	1.104	0.771	1.581
Presence of fever	0.974	0.822	0.236	2.649	0.529	1.250
Weight loss	0.073	0.077	0.345	1.075	0.925	1.250
Malaise	0.623	0.723	0.389	1.865	0.452	7.693
Arthralgia	-0.572	0.846	0.499	0.565	0.107	2.966
Myalgia	-1.454	0.930	0.118	0.234	0.038	1.446

## Discussion

This study highlights three important findings. First, antinuclear autoantibodies were more prevalent in pulmonary TB patients than in extrapulmonary TB patients. Second, the speckled ANA pattern was the most common pattern, observed in nine out of 13 ANA-positive patients, with six in pulmonary TB patients and three in extrapulmonary TB patients. Third, 92.3% of ANA-positive patients became negative after completing anti-TB therapy.

In our study, there was no significant difference in symptom duration between the ANA-negative (2.15 ± 1.77 months) and ANA-positive (1.77 ± 1.70 months) groups (p = 0.368). Weight loss was more pronounced in the ANA-positive group (6.00 ± 4.8 kg) compared to the ANA-negative group (4.80 ± 4.6 kg). Fever was the most common symptom in both groups (ANA-positive: 69.2%, ANA-negative: 68.6%). Our findings align with Elkayem et al., who also found fever to be the most common symptom, but we observed a shorter symptom duration (ANA-negative: 2.15 ± 1.77 months, ANA-positive: 1.77 ± 1.70 months) compared to their study (4.4 ± 1.7 months) [9].

Among 150 TB patients, 13 (8.7%) tested positive for ANAs, with 6/53 (11.3%) in pulmonary TB and 7/97 (7.21%) in extrapulmonary TB. Among extrapulmonary cases, tubercular lymphadenitis accounted for the majority, with 5/13 (38.5%) of ANA-positive results. Our findings align with a North Indian study reporting a similar ANA positivity rate (6.7%) in pulmonary TB [10]. In contrast, Shen et al. found a higher rate (32%) in pulmonary TB [6], while Jae et al. observed ANA positivity predominantly in extrapulmonary TB [11]. The most common ANA pattern observed was the speckled pattern, which was present in 9/13 (69%) ANA-positive patients. Of these, six were pulmonary TB cases, while three were extrapulmonary TB cases, all with tubercular lymphadenitis. Other extrapulmonary TB cases presented cytoplasmic or nucleolar patterns, whereas no pulmonary TB cases presented these patterns. These findings align with previous studies that identified the speckled pattern as the most common nuclear ANA fluorescence pattern [7,12].

In this study, most ANA-positive patients exhibited a moderate positive immunofluorescence (IF) pattern with one patient showing strong positive IF via the Hep-2 immunofluorescence technique. Notably, among the 13 ANA-positive patients, eight became ANA-negative within three months, and an additional four achieved ANA-negative status by the end of six months. These findings align with those of a previous study in which other autoantibodies, such as anticardiolipin IgG and anti-Scl-70, also returned to normal limits [6]. Importantly, the presence of these autoantibodies was not associated with any clinical signs of autoimmune diseases in active TB patients.

Elkayem et al. reported elevated anti-cyclic citrullinated peptide (CCP) autoantibodies in 32% of TB patients and increased IgM rheumatoid factor (RF) levels in 62% [9]. Kireev et al. found higher immunoglobulin and anti-tumor necrosis factor (TNF) autoantibody titers in TB patients compared to healthy donors, with disseminated TB cases showing higher TNF levels and IgG autoantibodies than localised TB cases [13].

Anti-citrullinated protein antibodies (ACPA) have been found in patients with tuberculosis, suggesting an autoimmune response, with all patients who were initially positive becoming negative after completing antituberculous treatment, indicating a link between these autoantibodies and active disease [14]. Serum levels of IFN-γ, TNF-alpha, and IL-10 were significantly elevated before treatment in tuberculosis patients and decreased significantly post-treatment [15]. Moreover, IFN-γ levels declined significantly over time in all TB patients, particularly in pulmonary TB patients during the first eight weeks of treatment, while no significant change was observed in extrapulmonary TB patients [16].

Notably, in our study, one patient continued to show strongly positive ANA test result even after completing anti-TB therapy. This patient, initially diagnosed with tubercular lymphadenitis, successfully completed her treatment without any complications. Given these findings, she was advised to follow up with a rheumatologist. ANA positivity can persist for a range of durations, from six to 48 months after completing anti-TB therapy, without causing clinical symptoms of autoimmune disease [11]. While anti-TB therapy can resolve some autoantibodies, it may also induce the formation of new autoantibodies, as observed with anti-lactoferrin and anti-myeloperoxidase (MPO) normalization, and the de novo formation of anti-PR3 and MPO antibodies [17]. Importantly, autoantibodies do not affect clinical progression, radiographic findings, or the risk of adverse events during active TB treatment. Elevated autoantibody levels normalize with anti-TB therapy alone, suggesting that these antibodies are likely a response to TB infection rather than an autoimmune disorder [6]. This highlights the importance of clinical correlation and ongoing follow-up to monitor such cases.

TB is a common cause of chronic inflammation and tissue damage, which can lead to the presence of autoantibodies in affected patients. In TB-endemic areas, if a patient has elevated autoantibodies without rheumatological symptoms, other potential causes, such as TB, should be considered, and sputum tests for acid-fast bacilli and mycobacterial culture are crucial. Failure to consider TB and misdiagnose patients with an autoimmune disease, followed by treatment with systemic corticosteroids, may increase the risk of disseminated TB and mortality [6]. This study did not find any significant associations between positive autoantibody status and clinicodemographic factors. Specifically, age, sex, and symptoms such as fever, malaise, rash, myalgia, and arthralgia did not significantly correlate with the ANA test results.

Strengths and limitations

The study’s strengths include its prospective design, a well-defined patient population, and longitudinal follow-up of ANA-positive cases with standardized testing, offering valuable insights from a TB-endemic region on a relatively understudied topic. However, the study has notable limitations, such as a small number of ANA-positive cases, the absence of a control group, and potential selection bias. The lack of a control group, including non-TB patients, limits the ability to draw definitive conclusions about whether ANA positivity is specifically linked to TB. Additionally, follow-up data were not collected for ANA-negative individuals, which may limit the understanding of dynamic changes in autoantibodies over time. While these limitations are acknowledged, further studies with a broader, more diverse population and a control group are necessary to better understand the relationship between ANA positivity and tuberculosis and to explore its potential clinical implications.

## Conclusions

Autoantibody tests, while valuable in identifying certain autoimmune diseases, can also yield positive results in various conditions, including infections like TB, without necessarily indicating an autoimmune disorder. Furthermore, TB patients may exhibit transient or low-level autoantibody positivity, which can complicate interpretation. Therefore, it is important to interpret autoantibody testing results cautiously and in conjunction with clinical assessment, microbiological investigations, and imaging studies to ensure a more accurate diagnosis and avoid misinterpretation. This holistic approach helps reduce the risk of diagnostic confusion and ensures that patients receive the most appropriate treatment based on their clinical presentation.
